# Modelo artesanal para treinamento de acesso vascular periférico

**DOI:** 10.1590/1677-5449.010216

**Published:** 2017

**Authors:** Ingrid Rodrigues de Oliveira Rocha, Monna Hessen Banna de Oliveira, Karolynie Lessa Bengtson, Antonio Márcio Nunes Alves, Marcus Vinícius Henriques Brito

**Affiliations:** 1 Centro Universitário do Estado do Pará – CESUPA, Laboratório de Cirurgia Experimental, Belém, PA, Brasil.; 2 Universidade do Estado do Pará – UEPA, Laboratório de Cirurgia Experimental, Belém, PA, Brasil.

**Keywords:** treinamento por simulação, vasos sanguíneos, desenvolvimento experimental

## Abstract

**Contexto:**

O acesso vascular é o procedimento mais comum realizado entre pacientes hospitalizados. Assim, na tentativa de minimizar complicações e aliar conhecimento técnico ao conhecimento teórico, os modelos de simulação são capazes de oferecer um ambiente seguro para profissionais em formação e evitar os dilemas éticos de treinamento direto em pacientes. Com esse objetivo, surgiram diversos manequins de treinamento, mas devido ao seu alto custo eles não são acessíveis a todos, e com frequência os profissionais em formação da área da saúde realizam procedimentos sem que tenham um treinamento prévio.

**Objetivo:**

Desenvolver um modelo de ensino e treinamento de acesso vascular periférico, utilizando um modelo de baixo custo para fins educacionais.

**Método:**

Para reproduzir a via periférica de acesso, utilizou-se um macarrão de polietileno com equipos de infusão, com uma extremidade em fundo cego e a outra conectada a duas bolsas de 500 mL de soro fisiológico acrescido de corante. A bolsa foi instalada em um suporte metálico.

**Resultado:**

O formato sugerido para o modelo apresentou semelhança com a anatomia do antebraço simplificada. O modelo se mostrou prático na punção e, devido à sua extensão, tem-se a possibilidade de puncionar diversas vezes o mesmo modelo, facilitando o treinamento.

**Conclusão:**

O modelo proposto permite o treinamento de acesso vascular periférico, sendo uma alternativa de baixo custo que pode ser utilizada para fins educacionais.

## INTRODUÇÃO

O acesso vascular é o procedimento mais comum realizado entre pacientes hospitalizados. É uma habilidade médica fundamental que exige de seu executor uma série de destrezas de cunho técnico e anatômico^1^. Procedimentos comuns incluem acesso venoso periférico para fins diagnósticos e terapêuticos, punção arterial nos procedimentos endovasculares e acessos cirúrgicos com abordagem vascular. No entanto, o acesso vascular pode proporcionar potenciais riscos e complicações, como infiltrações locais, formação de trombos, flebite, hematomas e sangramentos^2^^-^ 4.

Al-Elq^1^ afirma que os modelos de simulação são uma alternativa para minimizar essas complicações e aliar conhecimento técnico ao conhecimento teórico, além de serem capazes de oferecer um ambiente seguro para profissionais em formação e evitar os dilemas éticos de treinamento direto em pacientes ou em animais. O autor associou a simulação médica à possibilidade de uma aprendizagem eficaz e a um potencial para obter melhores resultados no manejo dos pacientes. Com esse objetivo, surgiram diversos manequins de treinamento, mas devido ao seu alto custo eles não são acessíveis a todos^5^.

Durante a formação acadêmica, os profissionais da área da saúde frequentemente realizam procedimentos, sejam eles ambulatoriais ou cirúrgicos, sem que tenham um treinamento prévio. Portanto, é comum que, pela falta de prática e pela influência de fatores psicológicos, ocorram falhas na execução desses procedimentos^6^.

Dessa forma, o objetivo deste trabalho foi desenvolver um modelo de ensino e treinamento de acesso vascular periférico, utilizando um modelo de baixo custo para fins educacionais.

## MÉTODOS

Para realização do modelo proposto, foram necessários os seguintes materiais (Tabela 1): macarrão de polietileno expandido de baixa densidade, de aproximadamente 45 cm; quatro equipos de soro; duas hastes de balão de festa; 20×40 cm de courvin; prancha de compensado; suporte metálico; braçadeiras plásticas; furadeira; soro fisiológico e corantes azul e vermelho.

**Tabela 1:** Lista de materiais utilizados.

**Material**
• Macarrão de polietileno expandido de baixa densidade (45 cm)
• Quatro equipos de soro
• Duas hastes de balão de festa
• 20×40 cm de tecido courvin
• Prancha de compensado (70×30 cm)
• Suporte metálico
• Cinco braçadeiras plásticas
• Soro fisiológico
• Corantes azul e vermelho
• Furadeira

**Figura 1:**
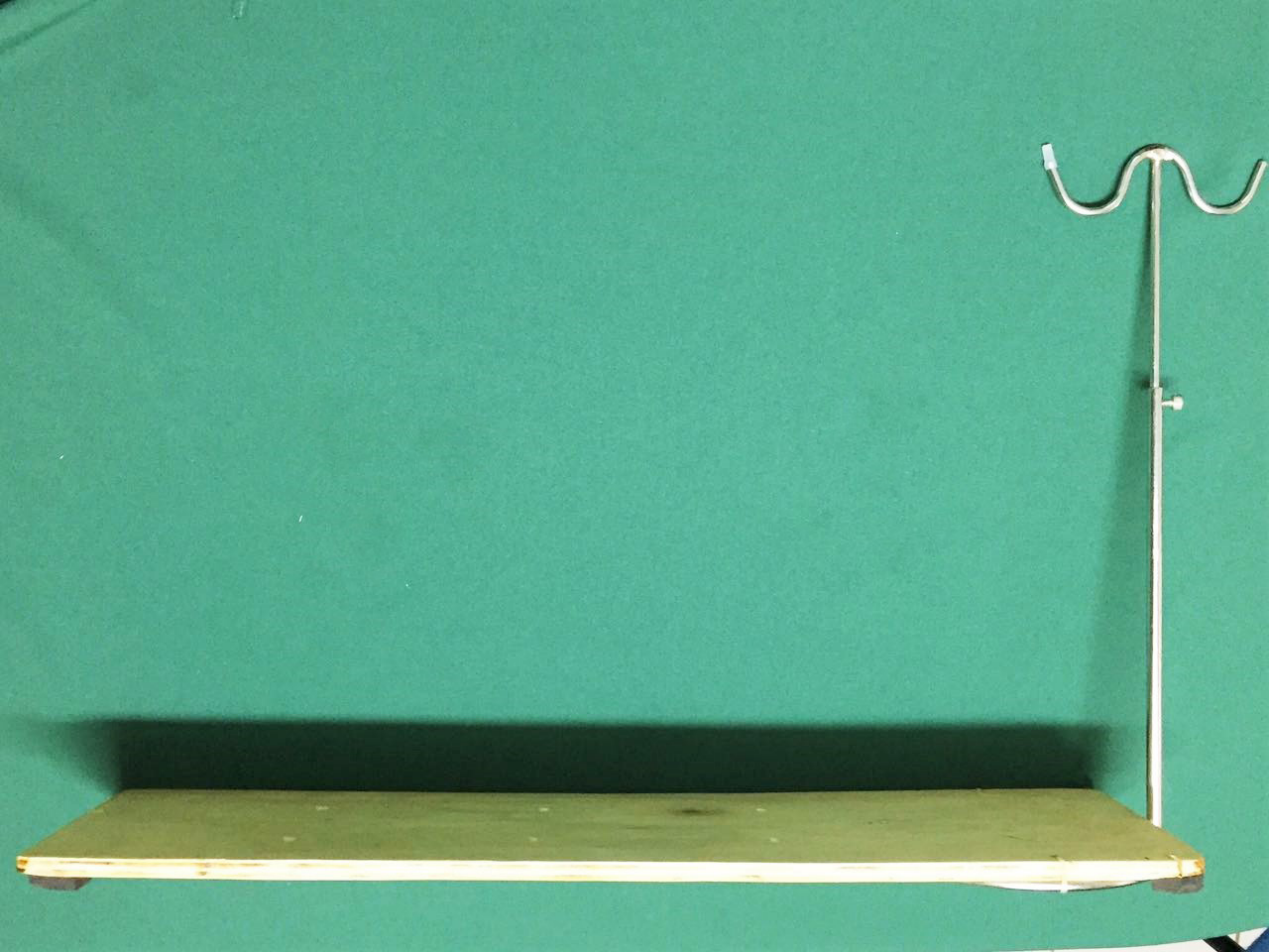
Finalização da base, com fixação do suporte de metal na prancha.

Para preparação do modelo, seguiram-se as seguintes etapas:

1. Confecção da base:

Com auxílio da furadeira, fez-se oito orifícios na prancha de compensado. Em seguida, procedeu-se à fixação do suporte metálico na prancha com duas braçadeiras plásticas inseridas nos orifícios laterais e presas na base do suporte (Figura 1).

**Figura 2:**
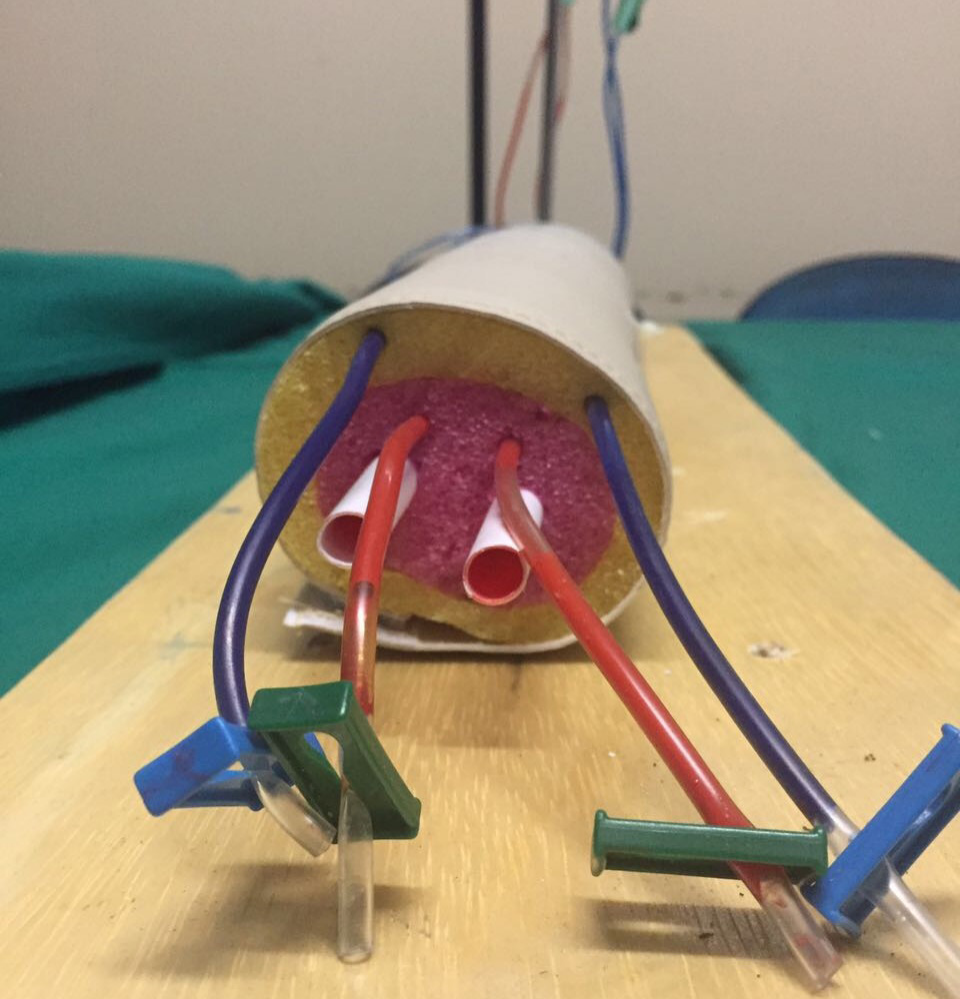
Visão transversal do macarrão de polietileno permitindo a visualização dos equipos de infusão e das duas hastes de balão de festa.

**Figura 3:**
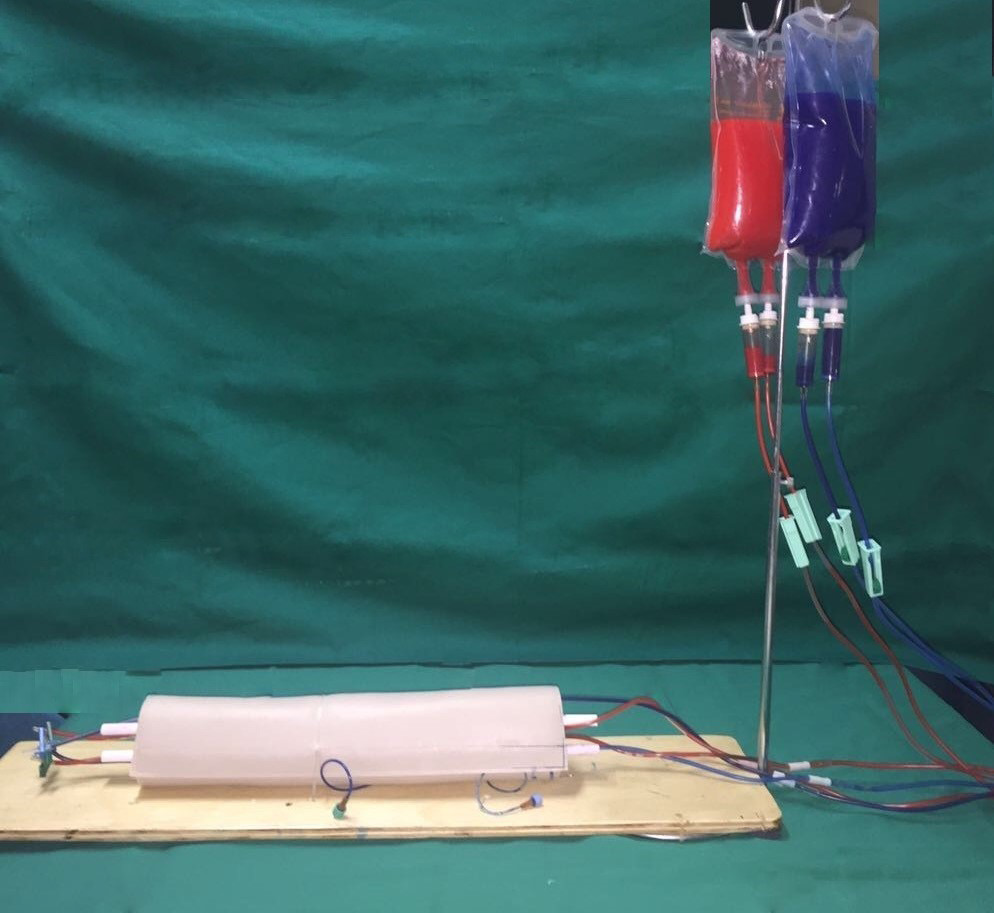
Resultado final: modelo sintético de antebraço.

1. Confecção do modelo:

O modelo foi feito a partir de 45 cm de macarrão de polietileno expandido de baixa densidade envolto por 20×40 cm de tecido courvin, para simulação do tecido muscular e pele respectivamente. Foram realizados orifícios no interior do macarrão de polietileno de modo a permitir a passagem de equipos de infusão e de duas hastes de balão de festa, em toda sua extensão longitudinal, a fim a simular a anatomia simplificada do antebraço (Figura 2). Nas bolsas de 500 mL de soro fisiológico foram acrescentados corante vermelho e azul para reproduzir didaticamente o sangue arterial o sangue venoso respectivamente, enquanto as hastes de balão de festa simularam os ossos rádio e ulna. A bolsa foi instalada no suporte de metal, facilitando a ação da gravidade (Figura 3). Por fim, o modelo foi fixado na base através das braçadeiras plásticas. A partir de então foi possível iniciar a prática do procedimento.

## RESULTADOS

**Figura 4:**
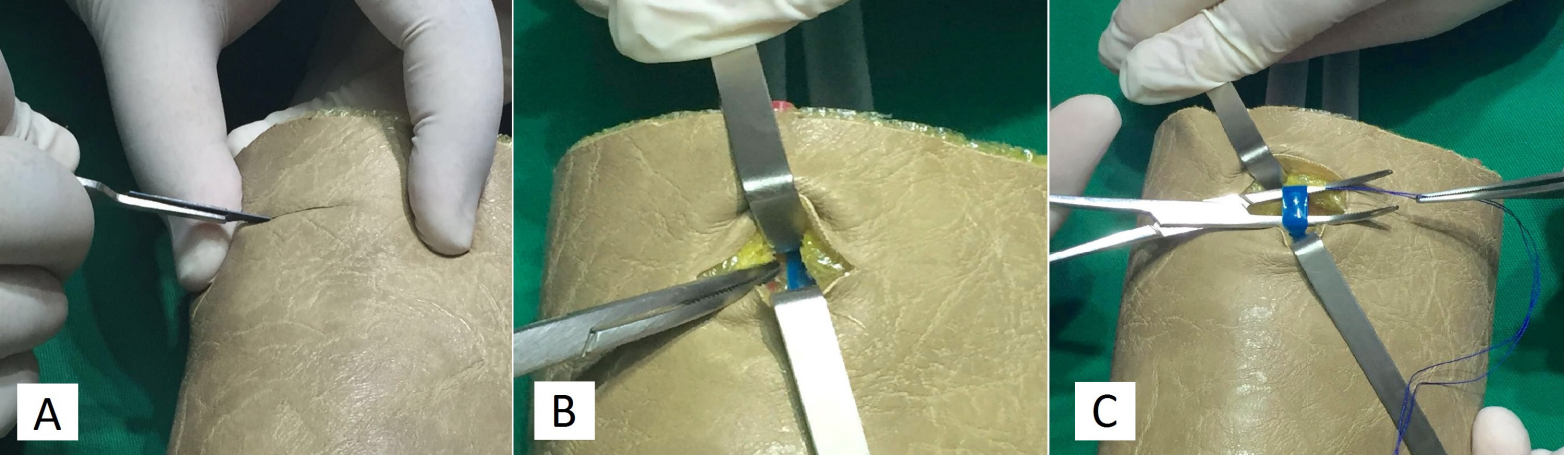
Treinamento e ensino dos acessos vasculares: (A) Criação da via de acesso na pele; (B) Exposição dos tecidos adjacentes; (C) Identificação do vaso sanguíneo e seu conteúdo líquido azul, representando o sistema venoso do antebraço.

O modelo criado apresentou configuração adequada para a representação mais realista dos tecidos humanos durante o ensino dos acessos vasculares, como a punção arterial e venosa e acessos cirúrgicos no membro superior (Figura 4A). O formato sugerido para o modelo apresentou semelhança com a anatomia normal do antebraço simplificada, identificando-se claramente os vasos sanguíneos, seu conteúdo líquido, os tecidos adjacentes e os planos teciduais (Figura 4B e C). O modelo se mostrou prático na punção e, devido à sua extensão, tem-se a possibilidade de puncionar diversas vezes o mesmo modelo, facilitando o treinamento.

## DISCUSSÃO

O acesso vascular é um procedimento que pode ser feito em diversos locais; porém, sua realização no braço e antebraço é a mais comum, pois estes possuem rica vascularização e são de fácil acesso. Vários fatores devem ser considerados para a realização do procedimento, como facilidade de inserção e acesso, tipo de agulha ou cateter a ser utilizado, bem como conhecimento da anatomia local. No presente modelo, achou-se necessário, além do treinamento da técnica, a reprodução da anatomia simplificada, visto que a região representada pelo modelo é território de veias e artérias importantes e muito utilizadas nas terapias endovenosas.

Inúmeros trabalhos na literatura referem que o índice de complicação é maior quanto menor for a experiência do operador, necessitando-se, portanto, de padronizações de treinamento para a adequada realização do acesso vascular. Para suprir a necessidade de destreza, foram desenvolvidos diversos modelos industriais de manequins de simulação humana, classificados em baixa, média e alta fidelidade. Este último tipo é caracterizado pelo alto custo de aquisição e necessidade de conhecimento avançado de operação técnica por parte de docentes e estudantes, e, ainda que representem aumento nos gastos em educação, essas tecnologias vêm ao encontro das expectativas de novas gerações de estudantes da área da saúde^6^^,^ 7.

O modelo proposto se assemelha com o manequim simulador anatômico do braço para acesso venoso comercializado pela indústria e disponível em várias marcas, com a vantagem de ser útil para a aquisição não somente da técnica de punção arterial e venosa como também de acessos vasculares para procedimentos cirúrgicos, noções de diérese em planos e treinamento de suturas. No contexto atual, a Educação Médica Baseada em Simulação (EMBS) já faz parte do currículo educacional de muitas universidades na América do Norte e Europa, e tal fato estimula diversas instituições de ensino a desenvolverem seus próprios simuladores, que permitam o treinamento e a aquisição do conhecimento a um custo mais baixo em relação aos disponíveis no mercado^1^^,^ 8.

O treinamento extensivo das habilidades práticas tem como objetivo seguir de forma simulada os mesmos passos aplicados na abordagem ao paciente e corrigir erros mais frequentes. A descrição desse modelo permite sua fácil reprodução, visto que os materiais usados na confecção são de fácil acesso e o método empregado na montagem é simples. Deve-se ressaltar, entretanto, que, o modelo serve como instrumento prático inicial. Para o aprimoramento da técnica, é fundamental a prática no paciente.

## CONCLUSÃO

O modelo proposto permite o treinamento de acesso vascular periférico, sendo uma alternativa de baixo custo, passível de confecção artesanal e que pode ser utilizada para fins educacionais.
