# Supplementary material for: Modelo artesanal para treinamento de acesso vascular periférico
Source: J Vasc Bras. 2017 Jul-Sep;16(3):195–8. [Article in Portuguese] doi: 10.1590/1677-5449.010216 (PMC5868934; doi:10.1590/1677-5449.010216)
Supplement: Translation

# Translation: Handmade model for peripheral vascular access training

**Authors:** Ingrid Rodrigues de Oliveira Rocha, Monna Hessen Banna de Oliveira, Karolynie Lessa Bengtson, Antonio Márcio Nunes Alves, Marcus Vinícius Henriques Brito

**Affiliations:** 1 Centro Universitário do Estado do Pará – CESUPA, Laboratório de Cirurgia Experimental, Belém, PA, Brazil.; 2 Universidade do Estado do Pará – UEPA, Laboratório de Cirurgia Experimental, Belém, PA, Brazil.

**Keywords:** training by simulation, blood vessels, experimental development

## Abstract

**Background:**

Vascular access is the procedure performed with greatest frequency in hospitalized patients. Simulation models are intended to minimize complications and combine technical and theoretical knowledge, offering a safe environment for training health professionals that avoids the ethical dilemmas of conducting initial training with patients. Many different training dolls have been developed to achieve this objective, but their high cost means they are not universally available, and it is common for unqualified health professionals to perform procedures on patients with no previous practice.

**Objective:**

To develop a low-cost educational model for teaching and training peripheral vascular access.

**Method:**

Peripheral access routes were reproduced using a polyethylene foam noodle and infusion kits, each with one extremity sealed off and the other connected to one of two 500 mL packs of saline, dyed red or blue. The packs were hung on a metal stand.

**Results:**

The structure chosen for the model was similar to a simplified version of the anatomy of the forearm. The model proved to be practical for puncture and, because of its length, the same model can be punctured multiple times, facilitating training.

**Conclusions:**

The model proposed here enables training of peripheral vascular access and is a low-cost option that can be used for educational purposes.

## INTRODUCTION

Vascular access is the procedure most frequently performed on hospitalized patients. It is a basic medical skill and its performance demands a series of technical and anatomical skills and dexterity.^1^ Common variants of the procedure include peripheral venous access for diagnostic and therapeutic reasons, arterial puncture for endovascular procedures, and surgical procedures via vascular access. However, vascular access can cause a series of potential risks and complications, such as local infiltration, formation of thrombi, phlebitis, hematoma, and bleeding.^2^^-^ 4

Al-Elq^1^ has stated that simulation models are one option for minimizing these complications and combining technical knowledge with theoretical knowledge, while offering a safe option for training health professionals that avoids the ethical dilemmas of direct training with patients or animals. This author associated medical simulation with the possibility of effective learning and the potential to achieve better results when managing patients. Many training dolls have been produced to achieve this objective, but their high cost means that they are not universally available.^5^

It is common for health professionals to perform clinical or surgical procedures during their academic courses with no prior training. It is therefore common that errors occur during these procedures, whether because of the lack of experience or due to the influence of psychological factors.^6^

In view of the above, this study was conducted with the objective of developing a low-cost teaching model for training peripheral vascular access.

## METHODS

The following materials were used to construct the model proposed (Table 1): approximately 45 cm of a low density expanded polyethylene foam noodle; four saline kits; two party balloon sticks; a 20×40 cm sheet of synthetic leather; a piece of laminate board; a metal drip stand; plastic cable ties; a drill; 2 saline packs; and blue and red dyes.

**Table 1:** List of materials used.

**Material**
• Low density expanded polyethylene foam noodle (45 cm)
• Four saline kits
• Two party balloon sticks
• 20×40 cm sheet of synthetic leather
• Laminate board (70×30 cm)
• Metal drip stand
• Five plastic cable ties
• Saline
• Blue and red dyes
• Drill

The following steps were taken to construct the model:

1. Preparation of the base:

The drill was used to make eight holes in the laminate board. The metal drip stand was then fixed to the board with two plastic cable ties inserted through holes at the side and fixed to the base of the drip stand (Figure 1).

**Figure 1:** Base prepared and metal stand fixed to board.

1. Construction of the model:

The model was constructed from a 45 cm length of low density expanded polyethylene foam noodle wrapped in a 20×40 cm piece of synthetic leather, to simulate the muscle tissue and skin, respectively. Holes were bored through the polyethylene noodle to allow the infusion kits and the two party balloon sticks to be advanced all the way through it longitudinally, simulating a simplified anatomy of the forearm (Figure 2). The 500 mL saline packs were colored red and blue to indicate arterial blood and venous blood respectively, while the balloon sticks represent the radius and ulna bones. The packs were hung on the metal support to facilitate the action of gravity (Figure 3). Finally, the model of the forearm was fixed to the base using plastic cable ties. It was then ready to be used to practice the procedure.

**Figure 2:** Cross-sectional view of polyethylene noodle showing infusion kits and two party balloon sticks.

**Figure 3:** Final result: synthetic model of the forearm.

## RESULTS

The model created offers an appropriate configuration to realistically represent human tissues during teaching of vascular accesses, such as arterial and venous punctures and surgical accesses via the upper limb (Figure 4A). The format proposed for the model exhibits similarity to the normal forearm anatomy, although simplified, clearly identifying blood vessels, their liquid contents, adjacent tissues, and tissue layers (Figure 4B and C). The model proved practical for puncture and, because of its length, the same model could be punctured several times, facilitating training.

**Figure 4:** Training vascular accesses: (A) Achieving access route in the skin; (B) Exposure of adjacent tissues; (C) Identification of blood vessel and its blue liquid content, representing the venous system of the forearm.

## DISCUSSION

Vascular access is a procedure that can be performed at many different sites, but the arm and forearm are the most common, because they are richly vascularized and offer easy access. Several factors should be considered in relation to the procedure, such as ease of insertion and access, type of needle or catheter to be used, and anatomical knowledge of the chosen site. It was decided that the model presented here should include a simplified reproduction of the anatomy rather than just the vessels, since the area represented is a territory in which there are important veins and arteries that are very often used for intravenous treatments.

Countless studies in the literature report that the rate of complications is greater, the lower the level of experience of the person performing the procedure, showing that there is a need to standardize training to ensure that vascular access is perforemd correctly. In order to meet the requirement for dexterity, several manufactured human simulation dolls have been developed and can be classified as low, medium, or high fidelity. High fidelty models are expensive to purchase and require advanced knowledge of their technical operation by both teachers and students and although they are responsible for increasing the cost of education, a new generatuion of students studying health-related subjects expect to have access to these technologies.^6^^,^ 7

The model proposed here is similar to commercially-available dolls that simulate the anatomy of the arm for venous access that are produced by several different brands and offers the advantage of utility not only for acquisition of arterial and venous puncture techniques, but also vascular accesses for surgical procedures, notions of dissection by layers, and suture training. Simulation-Based Medical Education is already part of the curicula at many universities in North America and Europe, which has encouraged many teaching institutions to develop their own simulators to enable training and knowledge acquisition at a lower cost than comercially available versions.^1^^,^ 8

Extensive training of practical skills aims to reproduce in a simulated manner the same steps employed when managing patients and correct the most common errors. The description of this model enables it to be easily reproduced, since the materials used to construct it are easily sourced and the construction method is simple. It should be emphasized, however, that this model is an initial practical instrument. To perfect the technique, practice with patients is of fundamental importance.

## CONCLUSIONS

The model proposed enables training of peripheral vascular access and is a low-cost alternative that can be made by hand and used for educational purposes.
